# Resection of liver metastasis from submandibular gland carcinoma five years after the primary operation: A case

**DOI:** 10.1016/j.amsu.2021.01.021

**Published:** 2021-01-19

**Authors:** Keigo Nakashima, Takeyuki Misawa, Yu Kumagai, Hiroaki Kitamura, Syuichi Fujioka, Katsuhiko Yanaga

**Affiliations:** a Department of Surgery, The Jikei University Kashiwa Hospital, 163-1 Kashiwashita Kashiwashi, Chiba, 277-8567, Japan; b Division of Digestive Surgery, The Jikei University School of Medicine, 3-19-18 Nishishinbashi Minatoku, Tokyo, 105-8471, Japan

**Keywords:** Hepatectomy, Liver metastasis, Submandibular gland carcinoma, Case report

## Abstract

**Introduction:**

Liver metastasis of submandibular gland carcinoma is not uncommon, yet its optimal management is still unclear. We report a case of resection of liver metastasis from submandibular gland carcinoma five years after the primary operation.

**Case presentation:**

The patient was a 76-year-old male who had undergone resection of primary adenoid cystic carcinoma of the submandibular gland in 2012. On follow-up computed tomography (CT) five years after the initial operation, a tumor was found incidentally in hepatic segment 6. Magnetic resonance imaging (MRI) confirmed the lesion's presence. Based on imaging findings and medical history, the lesion was suspected to be a liver metastasis of the previous submandibular gland carcinoma. The patient underwent hepatic posterior sectionectomy. His postoperative course was uneventful except for minor bile leakage that subsided without surgical intervention, and he was discharged on postoperative day 25. Postoperative pathological examinations of the hepatic tumor showed exactly the same features seen in the primary submandibular gland carcinoma, and the diagnosis as metastasis from this carcinoma was confirmed.

**Discussion:**

Liver resection may be a reasonable choice of treatment for liver metastasis of submandibular gland carcinoma. Further evidence from studies with larger patient populations must be accumulated to confirm this.

**Conclusion:**

We report our experience with a case of liver metastasis from submandibular gland carcinoma, which was resected five years after the primary operation.

## Highlights

• We report a case in which a tumor was found incidentally in hepatic segment 6.
• The submandibular gland was resected five years ago for adenoid cystic carcinoma.
• Postoperative pathological diagnosis was metastasis from the previous carcinoma.
• Liver resection may be useful for liver metastasis of submandibular gland carcinoma.

## 1 Introduction

Salivary gland carcinoma including submandibular gland carcinoma is rare [1,2]. Of reported cases of salivary gland carcinoma, adenoid cystic carcinoma (ACC) accounts for approximately 3–10% [3]. Because submandibular gland carcinoma may recur late, it has poor long-term prognosis [4]. Here, we report our experience with a rare case of liver metastasis from a submandibular gland carcinoma, which was resected five years after the primary operation. This work has been reported in line with the SCARE criteria [5].

## 2 Case presentation

**Fig. 1:**
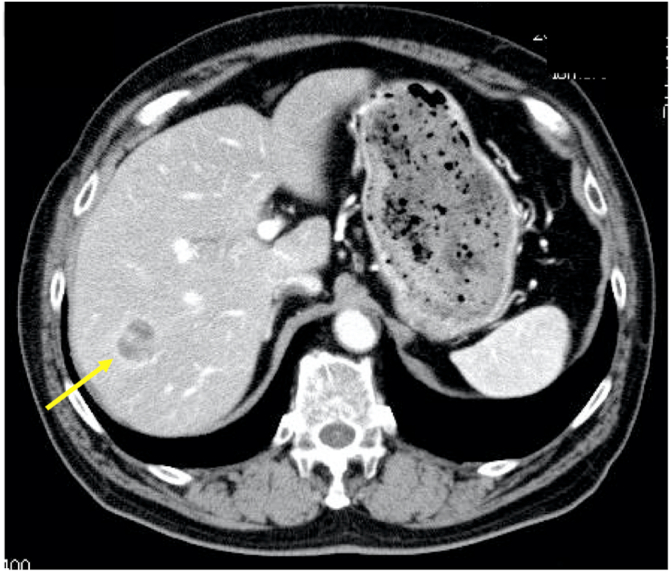
Preoperative CT A well-defined low density tumor with ring enhancement measuring 2.5 cm in diameter is seen in hepatic segment 6.

**Fig. 2:**
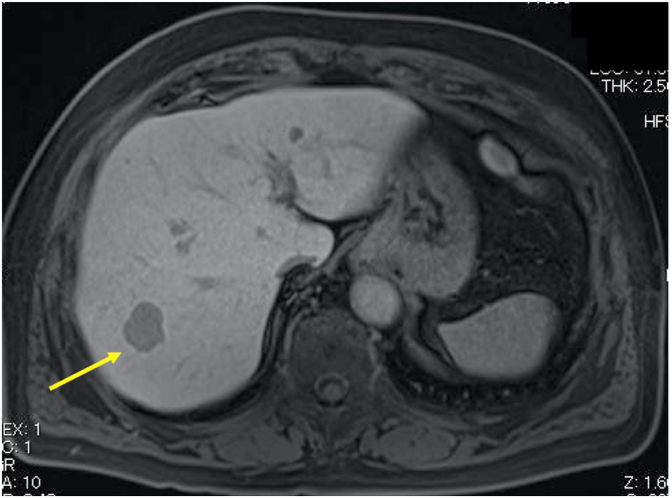
Preoperative CT A well-defined low density tumor with ring enhancement measuring 2.5 cm in diameter is seen in hepatic segment 6.

A male patient had undergone resection of the submandibular gland at the age of 71 in 2012; postoperatively, his condition was diagnosed as ACC, T2 N0 M0 Stage II. On follow-up computed tomography (CT) at the age of 76, five years after the initial operation, a well-defined low-density tumor with ring enhancement measuring 2.5 cm in diameter was incidentally found in hepatic segment 6 (Fig. 1). No local recurrence in other organs was found. Magnetic resonance imaging (MRI) confirmed the presence of the lesion (Fig. 2). Based on imaging findings and medical history, the lesion was suspected to be a liver metastasis of the previous submandibular gland carcinoma. The patient had a history of cholecystolithiasis, hypertension, and fatty liver but was otherwise healthy. Laboratory findings, listed in Table 1, included a slight elevation of serum CA19-9 and an indocyanine green (ICG) retention rate at 15 min of 19%.

**Table 1:** Laboratory data.

WBC	(/*μ* I)	8500	TP	(g/dl)	7.3
RBC	(× 10^6^/*μ* I)	5.05	Alb	(g/dl)	4.1
Hb	(g/dl)	15.5	Na	(mmol/dl)	141
Ht	(%)	47.8	K	(mmol/dl)	4.8
Plt	(× 10^3^/*μ* I)	187	CL	(mmol/dl)	103
AST	(IU/L)	50	CRP	(mg/dl)	0.2
ALT	(IU/L)	73	PT	(%)	100
LDH	(IU/L)	205	APTT	(sec)	28.9
T-Bil	(mg/dl)	0.7	CEA	(ng/ml)	2.6
BUN	(mg/dl)	15	CA19-9	(U/mol)	40
Cr	(mg/dl)	1.03	ICG R 15	(%)	19

**Fig. 3:**
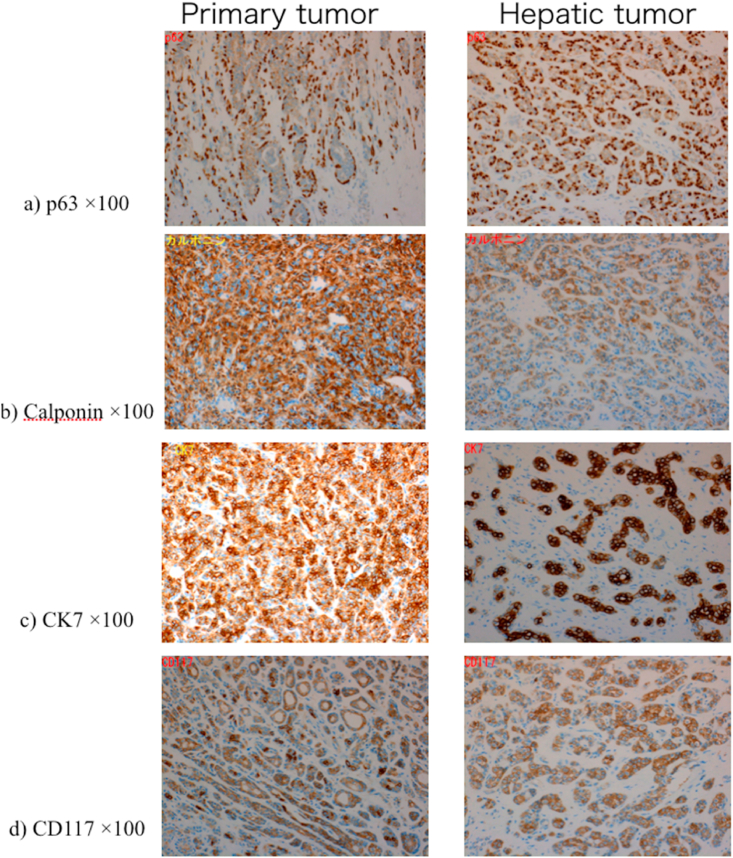
Pathological examinations Hepatic tumor cells were positive for P63, Calponin, CK7, and CD117. These features were the same as the primary submandibular gland carcinoma.

The patient underwent posterior sectionectomy of the liver with an operation time of 288 min and intraoperative blood loss of 150 ml. His postoperative course was uneventful except for minor bile leakage that subsided spontaneously, and he was discharged on postoperative day 25. Postoperative pathological examination revealed that the hepatic tumor was positive for P63, Calponin, CK7, and CD117 (Fig. 3). These features were exactly the same as those of the primary submandibular gland carcinoma; accordingly, the liver tumor was finally diagnosed as metastasis from the submandibular gland carcinoma (Fig. 3). The patient did not undergo postoperative adjuvant chemotherapy and remains free of disease and well as of six months after hepatic resection.

## 3 Discussion

Salivary gland tumors including submandibular gland tumors account for about 3–5% of all head and neck tumors [1,2]. Although ACC is the most common histopathological type of submandibular adenocarcinoma, it accounts for less than 10% of all neoplasms of the salivary gland [3]. Because submandibular gland carcinoma progresses slowly, its short-term prognosis is favorable, yet because delayed distant metastasis is not uncommon, its long-term prognosis is poor [4]. The five-year, 10-year, and 15-year survival rates are 70–75%, 37–69%, and 35–37%, respectively [[6], [7], [8]]. The incidence of metastasis of ACC of the submandibular gland is reported to be 47.1% [9], and the average time between primary diagnosis and the detection of metastasis is 36.8 months [10]. Metastasis can occur even a decade or more after the initial surgery at the primary site [11]. Distant metastases occur most often in the lungs (41–45%), followed by the brain (17–22%) and bone (10–15%). Liver metastasis, in contrast, is rare, with an incidence of only 4% [9].

Little has been published on liver resection for liver metastasis from submandibular gland carcinoma: only two cases were found in the PubMed database using a search for the keywords submandibular gland carcinoma, liver metastasis, and hepatectomy [12,13]. Perhaps because of its rarity, its optimal management is still unclear. Prognosis for patients with distant metastasis of submandibular gland ACC is generally poor: Sung et al. [14], for example, reported that 11% of patients with distant metastases died within one year after the diagnosis of metastasis while 33% died within three years. Long-term prognosis is poor, and radiotherapy and chemotherapy are not able to cure distant metastasis, either in the lung or elsewhere [15,16].

Surgery is known to be beneficial in cases of isolated lung metastasis of salivary grand carcinoma: several studies have reported a five-year postoperative survival rate of 84% [9,17]. For liver metastasis, therefore, liver resection may be a reasonable choice of treatment. Our patient had no concomitant medical conditions such as fatty liver or hypertension. When his solitary liver metastasis was diagnosed five years after his primary surgery, we decided to perform a hepatectomy. Because the effectiveness of adjuvant chemotherapy against metastatic ACC in terms of disease-free survival and overall survival remains unknown, we did not add adjuvant chemotherapy for our patient. Six months after surgery, this treatment appears to have been a success. Further evidence from studies with larger patient populations must be accumulated to confirm the usefulness of liver resection for liver metastasis of submandibular gland carcinoma.

## 4 Conclusion

We report our experience with a rare case of liver metastasis from submandibular gland carcinoma that was resected five years after the primary operation.

### 4.1 Patient perspective

The procedure of surgery was explained to the patient with all advantage and possible complications. He agreed on the procedure and informed consent was taken from him.

### 4.2 Informed consent

Written informed consent was obtained from the patient for publication of this case report and accompanying images. A copy of the written consent is available for review by the Editor-in-Chief of this journal on request.

## Ethical approval

Not applicable.

## Source of funding

The authors have no sponsors.

## Author contribution

KN wrote the manuscript. The remaining authors participated in revising the manuscript critically. TM, HK and SF performed the surgery. KY gave final approval of the manuscript. All authors read and approved the final manuscript for publication.

## Trial registration number

1 Name of the registry: Not applicable.

2 Unique Identifying number or registration ID:

3 Hyperlink to your specific registration (must be publicly accessible and will be checked):

## Guarantor

Keigo Nakashima.

## Declaration of competing interest

The authors declare that they have no conflicts of interest.
